# Effectiveness and Physiological Safety of a Lung Vibration Device for Airway Clearance in Patients with Sputum Retention: A Randomized Crossover Study

**DOI:** 10.3390/life16050794

**Published:** 2026-05-09

**Authors:** Tadsawiya Padkao, Kamolthip Channak, Panupich Kheunkhieo, Pimonpan Taweekarn, Kunavut Vannajak, Orachorn Boonla, Jatuporn Phoemsapthawee, Piyapong Prasertsri

**Affiliations:** 1 Faculty of Allied Health Sciences, Burapha University, Chonburi 20131, Thailand; tadsawiya@go.buu.ac.th (T.P.); pimonpan@go.buu.ac.th (P.T.); kunavut@go.buu.ac.th (K.V.); orachorn@go.buu.ac.th (O.B.); 2 Department of Rehabilitation, Namphong Hospital, Khon Kaen 40140, Thailand; nanptnu2527@gmail.com; 3 Department of Mechanical Engineering, Faculty of Engineering, Rajamangala University of Technology Isan, Khon Kaen Campus, Khon Kaen 40000, Thailand; panupich.kh@rmuti.ac.th; 4 Department of Sports Science, Faculty of Sports and Health Science, Kasetsart University, Nakhon Pathom 73140, Thailand; jatuporn.w@ku.th

**Keywords:** chest physiotherapy, airway clearance, lung vibration, sputum retention, respiratory rehabilitation, medical device, physiotherapy intervention

## Abstract

Lung vibration is a chest physiotherapy technique used to facilitate sputum mobilization and improve airway clearance; however, its effectiveness may vary due to therapist-dependent factors. This study developed a lung vibration device and evaluated its effectiveness in patients with sputum retention. Twenty-five patients, aged ≥18 years with sputum retention, including those with bronchiectasis, pneumonia, and COPD-related conditions, participated in a randomized crossover trial and received two single interventions in random order: a conventional intervention (manual percussion, manual vibration, and suction) and an experimental intervention (manual vibration replaced by the device). Sputum volume and quality, rating of perceived dyspnea (RPD), peripheral oxygen saturation (SpO_2_), cardiovascular dynamics, respiratory rate, and body temperature were assessed before and immediately after each intervention. Sputum volume was significantly higher following the experimental intervention compared with the conventional intervention (*p* = 0.010). No significant between-intervention differences were observed in sputum quality, RPD, SpO_2_, cardiovascular parameters, respiratory rate, or body temperature (all *p* > 0.05). No potential adverse effects were reported. These findings suggest that the lung vibration device enhances sputum clearance in the short term, with no immediate adverse physiological effects observed, and may serve as a practical alternative to manual vibration.

## 1. Introduction

Retention of airway secretions is a common clinical problem in patients with impaired respiratory function, including those with pulmonary disease, neuromuscular disorders, or limited mobility [1]. Inadequate sputum clearance can obstruct airflow, impair gas exchange, and increase the risk of pulmonary complications such as atelectasis and infection [2]. Chest physiotherapy is therefore widely employed to facilitate mucus mobilization and maintain airway patency [3].

Lung vibration is a frequently used chest physiotherapy technique that applies oscillatory forces to the chest wall, typically during expiration, to reduce sputum adherence and promote proximal movement of airway secretions [4]. When incorporated into standard airway clearance regimens alongside percussion and suctioning, lung vibration may enhance sputum removal and support ventilation [5]. However, manual lung vibration is inherently operator-dependent. Variations in applied force, vibration frequency, and treatment duration can lead to intra- and inter-therapist variability, potentially limiting treatment standardization and contributing to inconsistent clinical outcomes. Moreover, repetitive manual application may impose considerable physical demands on therapists, particularly in high-intensity care settings.

To overcome these limitations, mechanical or device-assisted lung vibration systems have been developed to deliver more consistent oscillatory stimulation [6]. Such devices may enhance treatment reproducibility while reducing therapist workload, particularly in small- to medium-sized hospitals where resources are limited. However, despite the availability of these devices, several gaps remain. Many commercially available systems are costly and may not be accessible in resource-limited settings, and there is limited evidence directly comparing device-assisted vibration with conventional manual techniques under clinically relevant conditions [6,7,8,9,10].

Accordingly, the novelty of the present study lies in three key aspects: (1) the development of a locally designed, cost-effective lung vibration device suitable for routine clinical use; (2) its direct comparison with manual lung vibration using a randomized crossover design; and (3) its evaluation in an inpatient setting, thereby enhancing the clinical relevance and applicability of the findings.

Therefore, this study aimed to develop an innovative lung vibration device and evaluate its effectiveness compared with manual chest wall vibration as part of standard chest physiotherapy in patients with sputum retention. In a randomized crossover design, sputum quantity and quality, perceived dyspnea, oxygen saturation, and cardiovascular parameters were assessed before and after each intervention to determine whether the device provides a safe and clinically comparable alternative to manual lung vibration.

## 2. Materials and Methods

### 2.1. Lung Vibration Device

#### Innovation of the Lung Vibration Device

The lung vibration device was developed by a mechanical engineer (P.K.) with expertise in medical device and robotic innovation. The device is constructed from durable, nonconductive plastic to enhance safety and minimize the risk of electrostatic discharge. It features a rectangular base with rounded edges and an integrated handle to ensure safe and ergonomic operation. The overall dimensions are 185 × 110 × 215 mm, with a contact surface area of 180 × 110 mm, and a total weight of approximately 1.2 kg. A black silicone-covered head is securely attached to the base to facilitate contact with the chest wall.

**Figure 1:**
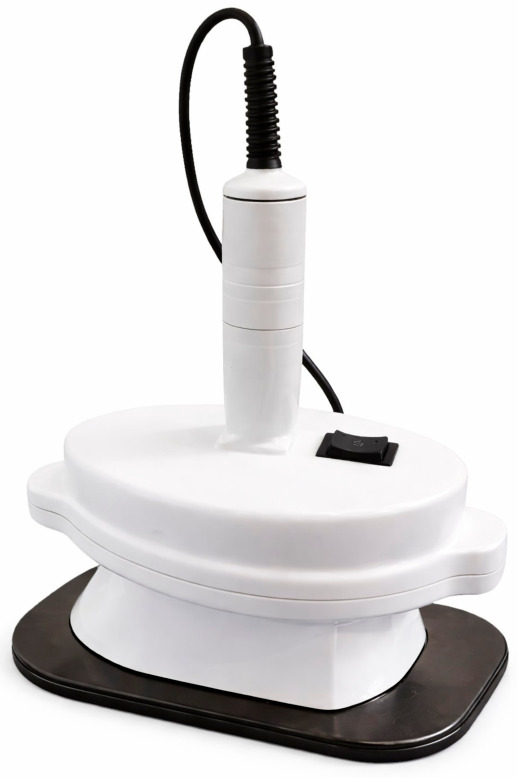
The innovative lung vibration device.

The device operates on a 220 V power supply (50–60 Hz) with a power consumption of 22 W. A built-in power switch allows convenient activation. Vertical vibrations are generated by an internal motor, producing a controlled percussive force applied to the chest wall at a frequency of approximately 2700 revolutions per minute (≈45 Hz) (Figure 1).

### 2.2. Effectiveness Evaluation of Lung Vibration Device

#### 2.2.1. Study Design and Sample Size

This study employed a randomized crossover design to evaluate the effectiveness of a lung vibration device. A total of 25 patients aged ≥18 years with respiratory problems and mucus retention were included.

The sample size was calculated based on a previous randomized crossover study by Murray et al. [11], which investigated airway clearance in patients with bronchiectasis (*n* = 20) and reported significant changes in sputum volume. Sample size estimation was performed for a paired comparison (two dependent means) using G*Power software (version 3.1.9.4; Universität Kiel, Germany), assuming an effect size of approximately 0.73, a statistical power of 0.80, and an α-error probability of 0.05. The minimum required sample size was 22 patients. To account for an anticipated dropout rate of approximately 10%, the final target sample size was increased to 25 patients.

Patients and the public were not involved in the design, conduct, reporting, or dissemination of this trial.

#### 2.2.2. Patient Recruitment and Screening

Patient recruitment and screening were conducted at Namphong Hospital, Namphong District, Khon Kaen Province. This study was approved by the Human Research Ethics Committee of Burapha University (IRB approval number: IRB1-014/2568; approval date: 7 February 2025) and the Khon Kaen Provincial Public Health Office. The trial was prospectively registered at ClinicalTrials.gov (Identifier: NCT07056764; registration date: 8 July 2025).

Patients were selected based on the presence of clinically significant pulmonary mucus retention requiring airway clearance therapy, rather than disease-specific diagnoses. Although recruitment was based on this functional clinical condition, underlying medical diagnoses were recorded and are presented in Table 1 to provide clinical context.

Inclusion criteria were as follows: (a) male or female patients aged ≥18 years; (b) inpatients at Namphong Hospital with documented pulmonary mucus retention; (c) independence from ventilatory support; and (d) referral by the attending physician for enhanced mucus clearance via physiotherapy. Exclusion criteria, applied to ensure patient safety during the early-stage evaluation of this novel device, included: (a) inflamed wounds on the chest or back; (b) unstable vital signs, defined as resting heart rate (HR) <60 or >100 bpm, blood pressure <90/60 or >140/90 mmHg, respiratory rate (RR) <12 or >20 /min, peripheral oxygen saturation (SpO_2_) < 95%, or body temperature (BT) > 37 °C; (c) bleeding tendency, defined as a platelet count < 20,000/mm^3^; (d) use of medications associated with increased bleeding risk within 7 days prior to participation, including anticoagulant or antiplatelet agents; (e) international normalized ratio > 1.1 in patients not receiving anticoagulants, or >3.0 in those receiving anticoagulant therapy; (f) subcutaneous emphysema; (g) severe hemoptysis; (h) chest wall pain; (i) acute spinal cord injury, rib fracture, or conditions associated with a high risk of bone fracture; (j) skin grafts or burn wounds; (k) admission to the intensive care unit (ICU), respiratory ICU, or cardiac ICU; (l) untreated pneumothorax; (m) acute inflammatory pulmonary conditions; (n) active respiratory infections, including tuberculosis, lung abscess, or COVID-19; and (o) respiratory diseases contraindicated for lung vibration therapy, including severe or unstable respiratory conditions as determined by the physician.

In routine clinical practice, airway clearance techniques are often applied to patients with broader ranges of vital signs, including those with mild tachycardia, tachypnea, or reduced oxygen saturation, with appropriate monitoring and clinical judgment [12,13,14]. This reflects the use of individualized clinical decision-making rather than strict physiological cut-off thresholds. However, the present study employed more conservative thresholds to ensure patient safety during early-stage device evaluation.

#### 2.2.3. Treatment Randomization and Intervention Procedures

**Figure 2:**
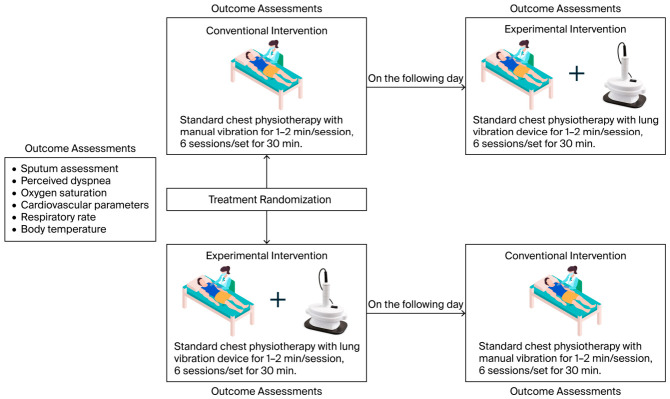
Study intervention procedures, illustrating randomization, interventions, and the assessment timeline.

Following the screening process, written informed consent was obtained from each patient or their legal representative. All patients received both interventions (conventional and experimental) in a randomized crossover sequence (Figure 2) using simple randomization. Patients were assigned to one of two sequences: conventional–experimental or experimental–conventional. Outcomes were assessed separately for Period 1 and Period 2 to evaluate potential order effects.

The allocation sequence was generated by the principal physical therapist (K.C.) and implemented at the time of intervention assignment. Due to the crossover design, all patients received both interventions in randomized order, thereby minimizing allocation bias.

In the conventional intervention, patients received standard chest physiotherapy, including manual chest wall percussion, manual vibration (at a frequency of approximately 10.5 Hz), and suctioning. Manual chest vibration was applied for 1–2 min per session, repeated continuously for at least six sessions per treatment set, with a total treatment duration not exceeding 30 min.

**Figure 3:**
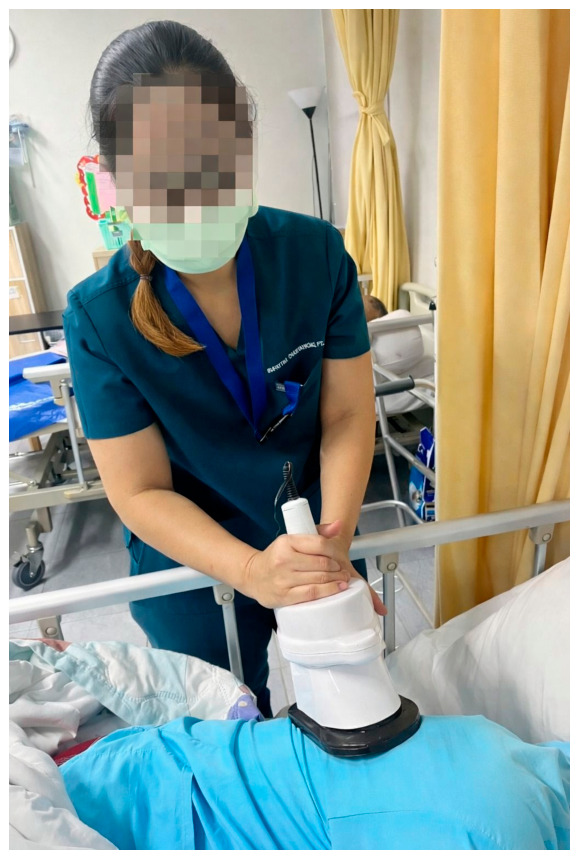
Example of the application of the lung vibration device in a patient.

In the experimental intervention, patients received standard chest physiotherapy in which manual chest vibration was replaced by a mechanical lung vibration device (at a frequency of approximately 45 Hz) (Figure 3), while chest percussion and suctioning were maintained. The lung vibration device was applied for 1–2 min per session, repeated continuously for at least six sessions per treatment set, with a total treatment duration not exceeding 30 min.

Both interventions were delivered by a licensed physical therapist with expertise in cardiopulmonary rehabilitation. To ensure consistency and minimize inter-operator variability, all procedures were performed by the same therapist, who underwent standardized training and adhered to predefined intervention protocols throughout the trial.

Sputum quantity and quality, rating of perceived dyspnea (RPD), SpO_2_, cardiovascular dynamics, RR, and BT were assessed before and after each intervention. On the following day, patients crossed over to the alternate intervention: those who initially received the conventional intervention subsequently received the experimental intervention, and vice versa.

No washout period was implemented due to the clinical necessity of daily airway clearance therapy. Given the short-term and immediate nature of the intervention effects, carryover effects were considered unlikely, although they cannot be entirely excluded.

Patients were withdrawn from the study if any of the following occurred: abnormal reactions during treatment (e.g., pain at the device application site, headache, nausea, chest tightness or pain, dyspnea, hyperpnea, hypertension, or SpO_2_ < 95%); unstable vital signs during the intervention; signs suggestive of internal bleeding within 2 h after the first treatment (e.g., blood-streaked sputum, petechiae, or bruising on the chest or back); changes in medication regimen during the study period; or voluntary withdrawal of consent.

Due to the nature of intervention, patients and the physical therapist delivering the interventions were not blinded. Outcome measures were assessed using standardized procedures, and data analysis was performed independently to minimize potential bias.

### 2.3. Study Outcomes and Assessments

The primary outcome of this study was sputum volume measured immediately after the intervention. Secondary outcomes included sputum quality, RPD, SpO_2_, cardiovascular parameters, RR, BT, and potential adverse effects.

#### 2.3.1. Sputum Assessment

After each intervention, sputum quantity was assessed using a sterile transparent graduated container and documented photographically. To enhance consistency, all sputum collection procedures were performed by the same therapist using standardized suction techniques immediately after intervention.

Suctioning was conducted using a sterile catheter with controlled negative pressure (100–120 mmHg) and standardized duration, in accordance with current clinical practice recommendations for airway suctioning and established physiotherapy procedures for airway clearance [14,15,16]. Each suction episode was limited to a brief duration to minimize patient discomfort and procedural variability, and all procedures were performed within a standardized daytime period (9:00 AM–3:00 PM). During the sputum collection period, mucolytic medications were withheld, while all other prescribed treatments were maintained. Sputum was collected into a sterile graduated container, and care was taken to minimize contamination from upper airway secretions or saliva. Only sputum obtained through suctioning and considered representative of lower airway secretions was included in the analysis. In addition, sputum quality was evaluated using a standardized sputum quality assessment form [17], with minor modifications, which classified sputum according to type (mucoid, mucopurulent, or purulent) and pourability (graded from 1 to 4, ranging from adherent to easily pourable). Photographic documentation was used to support classification and verification.

All procedures were conducted under similar clinical conditions, with efforts made to maintain consistency in patient positioning and timing relative to the intervention [12,14,16]. Nevertheless, sputum volume measurement may still be influenced by factors such as suction duration, hydration status, and secretion source (upper vs. lower airway), and should be interpreted as a pragmatic clinical measure.

Hydration status was managed under physician supervision as part of routine clinical care, with fluid intake individualized based on each patient’s body weight and/or body surface area to ensure adequate hydration. This approach is consistent with standard clinical practice in hospitalized patients, where fluid therapy is adjusted to maintain physiological stability [18,19].

#### 2.3.2. Dyspnea Assessment

Dyspnea was evaluated using the RPD scale, corresponding to the Borg 6–20 scale, with scores ranging from 6 (no dyspnea) to 20 (maximal dyspnea). The RPD score was recorded in a resting condition before and after each intervention session.

#### 2.3.3. Peripheral Oxygen Saturation Assessment

SpO_2_ was assessed using a standard hospital-grade pulse oximeter, with %SpO_2_ obtained from the index finger. Measurements were performed while patients were in a resting condition immediately before and after the intervention, and the recorded value represented a stable reading free of motion artifacts.

#### 2.3.4. Cardiovascular Dynamics, Respiratory Rate, and Body Temperature Assessments

Systolic and diastolic blood pressure (SBP and DBP) and HR were measured under resting conditions before and after the intervention using a bedside BP monitor. Mean arterial pressure (MAP), pulse pressure (PP), and rate–pressure product (RPP) values were subsequently calculated using the following equations [20]: PP (mmHg) = SBP − DBP; MAP (mmHg) = DBP + 1/3 × PP; and RPP (mmHg·bpm) = SBP × HR. RR was measured using a manual method, while BT was assessed using a digital thermometer.

#### 2.3.5. Potential Adverse Effects Assessment

Breathlessness and chest pain were also assessed after each intervention as indicators of potential adverse effects, with particular attention to the experimental intervention involving the lung vibration device.

### 2.4. Data Analyses

All statistical analyses were conducted using IBM SPSS Statistics for Windows, version 25.0 (IBM Corp., Armonk, NY, USA). Continuous variables are presented as mean ± standard deviation (SD), unless otherwise stated. Normality was assessed using the Shapiro–Wilk test.

Within-intervention comparisons (pre- vs. post-intervention) were performed using paired *t*-tests. Between-intervention differences were analyzed using a linear mixed-effects model with fixed effects for treatment, period, and sequence, and participant included as a random effect. This model accounts for within-subject correlations and enables estimation of treatment effects while adjusting for potential period and sequence effects. Sputum type was categorized descriptively as mucoid, mucopurulent, or purulent and coded as ordinal values of 1, 2, and 3, respectively, for analysis. Statistical significance was set at *p* < 0.05.

No interim analyses or predefined stopping rules were applied. As no missing data were observed, no imputation procedures were required. Additionally, no subgroup, sensitivity, or post hoc analyses were conducted beyond the prespecified statistical analysis plan.

### 2.5. Changes to Trial Protocol

No important changes to the trial protocol, prespecified outcomes, or statistical analysis plan were made after trial commencement.

## 3. Results

**Figure 4:**
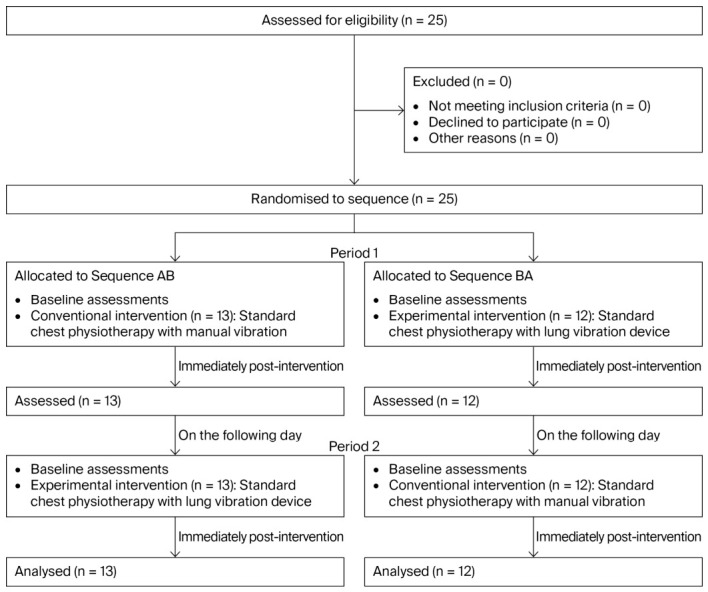
CONSORT flow diagram of patient enrollment, allocation, follow-up, and analysis. A total of 25 patients were assessed for eligibility, of whom 25 met the inclusion criteria and were randomized into two sequences: Sequence AB (conventional intervention followed by experimental intervention) and Sequence BA (experimental intervention followed by conventional intervention). In Period 1, 13 patients received the conventional intervention and 12 received the experimental intervention. All patients completed Period 1 and crossed over to the alternate intervention on the following day (Period 2). In Period 2, 13 patients received the experimental intervention and 12 received the conventional intervention. All randomized patients completed both intervention periods and were included in the final analysis.

The trial was conducted between August 2025 and January 2026. Patients were recruited during this period, and outcome assessments were performed before and after each intervention session. A total of 25 patients were included and randomized to receive two interventions in a crossover design. In the first period, 13 patients received the conventional intervention, while 12 received the experimental intervention. All patients completed both study periods, and no patients were excluded. Data from all patients were included in the final analysis (Figure 4).

### 3.1. Baseline Clinical Characteristics of Patients

Table 1 summarizes the baseline clinical characteristics of the patients. Most patients were female (76%), with a mean age of 71.21 years and a mean body mass index of 24.97 kg/m^2^. Approximately 40% required supplementary oxygen, and the mean hospital stay was 8.76 days. The majority were non-smokers (80%) and received bronchodilator therapy (76%), whereas smaller proportions received antibiotics (32%) and corticosteroids (4%). The most common diagnoses were bronchiectasis (44%) and pneumonia (32%), followed by COPD with bronchiectasis (20%) and COPD with respiratory failure (4%).

### 3.2. Sputum Quantity and Quality

Following the intervention, mean sputum volume was significantly higher in the experimental condition than in the conventional condition (4.69 ± 2.51 mL vs. 3.31 ± 1.39 mL; mean difference, +1.38 mL; *p* = 0.005). In contrast, sputum type did not differ significantly between conditions (*p* = 0.107). The mean pourability score was 2.64 ± 0.61 in the experimental condition and 2.44 ± 0.56 in the conventional condition, showing a non-significant trend toward higher scores in the experimental group (mean difference, +0.20; *p* = 0.061) (Table 2). No significant period or sequence effects were observed for any outcome (Table 3).

### 3.3. Dyspnea Score and Peripheral Oxygen Saturation Level

No significant within-group changes in RPD scores were observed in either the conventional (*p* = 0.241) or experimental (*p* = 0.173) condition. In contrast, SpO_2_ increased significantly in the experimental condition (*p* < 0.001). However, between-condition comparisons showed no significant differences in either RPD scores or SpO_2_ levels (*p* = 0.357 and *p* = 0.701, respectively) (Table 2). No significant period or sequence effects were detected for any outcome (Table 3).

### 3.4. Cardiovascular Dynamics, Respiratory Rate, and Body Temperature Level

Following the conventional intervention, HR and RPP increased significantly (both *p* < 0.001). Similarly, the experimental intervention resulted in significant increases in HR (*p* < 0.001), SBP (*p* = 0.003), PP (*p* = 0.007), and RPP (*p* < 0.001). No significant changes were observed in DBP, MAP, RR, or body temperature after either intervention.

Between-condition comparisons showed no significant differences in any cardiovascular parameters, RR, or body temperature (Table 4). No significant sequence effects were observed for any outcome. However, a significant period effect was detected for HR (*p* = 0.016) and PP (*p* = 0.037) (Table 5).

### 3.5. Potential Adverse Effects

Potential adverse effects were assessed systematically following each intervention session using standardized observation and patient self-report. Predefined criteria were used to identify adverse events, and patients were monitored throughout and immediately after each intervention. No patients reported breathlessness or chest pain following either the conventional (0%) or experimental intervention (0%), indicating the absence of observed adverse effects (Table 6).

## 4. Discussion

This randomized crossover study evaluated the effectiveness and safety of a lung vibration device as an alternative to manual vibration in chest physiotherapy for patients with sputum retention. The main finding was that the experimental intervention significantly increased sputum volume compared with the conventional approach, while producing comparable effects on sputum characteristics, RPD, SpO_2_, and physiological parameters.

The study population included patients with bronchiectasis, pneumonia, and COPD-related conditions, which are commonly associated with mucus retention and impaired airway clearance. These conditions are characterized by excessive mucus production, altered mucociliary transport, and increased risk of secretion accumulation within the airways [1,2,21]. This supports the clinical relevance of the present findings, as airway clearance techniques are routinely applied in these populations to facilitate mucus mobilization, improve ventilation, and reduce the risk of respiratory complications [3,12]. Therefore, the inclusion of these patient groups enhances the external validity of the study in real-world clinical practice, particularly for clinically stable hospitalized patients with mucus retention requiring airway clearance therapy, rather than for specific disease categories or critically ill populations.

The greater sputum volume observed with the device may suggest enhanced short-term airway secretion mobilization; however, its clinical significance remains uncertain. This effect may be partly explained by the device’s ability to deliver consistent and reproducible oscillatory mechanical forces, thereby reducing the intra- and inter-therapist variability inherent in manual techniques [22,23]. In addition, the higher vibration frequency delivered by the device (approximately 45 Hz), compared with manual vibration (approximately 10.5 Hz), may further contribute to improved mucus detachment and transport. This difference in vibration frequency may also contribute to the observed effect on sputum clearance by promoting greater mucus detachment and transport. Mechanical vibration also provides controlled frequency and amplitude stimuli to the thorax, which may facilitate sputum loosening and mobilization. These features, together with the non-invasive nature and high reproducibility of the device, support its potential utility in clinical settings [6].

Despite the increased sputum volume, sputum characteristics (mucopurulent) and pourability did not differ significantly between interventions. This suggests that the device primarily enhances secretion clearance rather than modifying mucus properties during a single treatment session. This may indicate that the overall mechanical stimulus applied to the thorax was functionally comparable between manual and device-assisted techniques in terms of effects on mucus rheology. However, the non-significant trend toward greater pourability in the experimental condition (+0.20, p = 0.061) may indicate a modest effect that warrants further investigation in studies with larger sample sizes or repeated interventions to determine whether cumulative exposure to device-based vibration can meaningfully influence sputum properties.

The present study should be interpreted as a comparison between two airway clearance approaches with different mechanical characteristics, rather than a direct comparison of manual versus device-assisted delivery of an identical vibration stimulus. Manual chest percussion and vibration are typically delivered at lower frequencies, approximately 5–11 Hz [22,23,24], whereas high-frequency chest wall oscillation protocols generally operate within a range of 5–20 Hz [6,12]. In contrast, the device used in this study delivered vibration at approximately 45 Hz, which may have contributed to the greater sputum volume observed following the experimental intervention. Therefore, the observed effect is likely attributable not only to improved consistency and reproducibility but also to differences in vibration frequency and intensity.

Although sputum volume increased, this outcome is inherently variable and influenced by both procedural and physiological factors. The relatively large standard deviations indicate considerable inter- and intra-individual variability. This variability is expected, as sputum production is affected by multiple factors, including disease severity, hydration status, and airway responsiveness. Moreover, an increase in sputum volume immediately after treatment does not necessarily translate into clinically meaningful benefits, such as symptom improvement, reduced complications, or enhanced respiratory function.

Adequate hydration is known to influence mucus rheology and airway secretion transport, as airway surface liquid volume plays a critical role in maintaining effective mucociliary clearance. Dehydration may increase mucus viscosity and impair clearance, whereas sufficient hydration helps maintain mucus mobility and facilitates secretion removal [25]. In the present study, hydration was maintained at clinically appropriate levels to minimize its potential influence on sputum characteristics.

No significant changes in dyspnea scores were observed following either intervention, likely reflecting the short assessment period and the relatively stable clinical status of the patients. Although SpO_2_ increased significantly within the experimental intervention (+3.19%), the absence of between-intervention differences suggests that both approaches were comparably effective in maintaining oxygenation [26,27]. These findings are partly consistent with previous reports indicating that airway clearance techniques can enhance secretion removal without necessarily producing immediate, perceptible improvements in dyspnea [28]. Variability across studies may be attributed to heterogeneity in intervention protocols, including session duration and treatment intensity. Furthermore, studies demonstrating significant reductions in dyspnea often involve patients with higher baseline symptom severity than those included in the present study [29]. Notably, greater improvements in dyspnea have been reported when airway clearance techniques are combined with structured breathing techniques [30].

Both interventions elicited mild increases in cardiovascular parameters, particularly HR and RPP (+6.83 vs. +7.15 bpm and +10.51 vs. +15.09 mmHg·bpm in the conventional and experimental interventions, respectively), indicating an acute physiological response to chest physiotherapy [26,27]. Additional increases in SBP (+6.19 mmHg) and PP (+5.33 mmHg) were observed following the experimental intervention, while other parameters remained stable. These changes may be related to the higher vibration frequency applied in the device-based intervention, which could induce slightly greater hemodynamic, respiratory, and metabolic demand [31]. However, no significant between-intervention differences were detected. Collectively, these findings support that the lung vibration device does not impose additional cardiovascular or respiratory burden compared with conventional therapy [7,32,33].

Importantly, no adverse effects, including breathlessness or chest pain, were reported following either intervention, suggesting that both approaches were well tolerated under the study conditions [12,21], particularly in older patients with potential comorbidities. Owing to the crossover design, the overall treatment components and applied forces were comparable between interventions, with the primary distinction being the vibration frequency. Therefore, both standard chest physiotherapy using manual vibration and its substitution with the lung vibration device did not appear to exacerbate patient symptoms or induce unintended clinical effects. However, this observation is limited to immediate responses following single-session interventions in a small clinically stable population. Accordingly, conclusions regarding safety should be interpreted with caution, and further studies are required to evaluate long-term safety and effects in broader patient populations.

The present study has several limitations. First, although the sample size was calculated a priori, the relatively small number of patients may have limited statistical power for detecting differences in some outcomes. In addition, the single-session design precludes conclusions regarding long-term clinical benefits; thus, future studies with larger samples and extended intervention periods are warranted to enhance generalizability. Second, while a key strength of this study is the use of a crossover design and a single physiotherapist to minimize inter-individual and operator variability, the absence of blinding may have introduced potential bias. Third, the heterogeneous nature of the underlying diagnosis may have introduced variability in treatment response, as different respiratory conditions may present with distinct mucus characteristics and airway dynamics. In addition, the selective inclusion of clinically stable patients and exclusion of more severe or unstable conditions may limit the generalizability of the findings to broader patient populations. These relatively strict physiological criteria may further limit the applicability of the findings to patients with more variable or clinically unstable vital signs commonly encountered in routine practice. Although patients had heterogeneous underlying diagnoses, strict exclusion criteria were applied to ensure safety during early-stage device evaluation. As a result, the study population represents clinically stable patients with sputum retention, and findings may not generalize to more severe or unstable populations. Fourth, safety was evaluated only immediately after single intervention sessions using basic physiological parameters and self-reported symptoms, which limits the ability to draw conclusions regarding long-term safety. Finally, objective assessments of respiratory function (e.g., FEV_1_, FEV_1_/FVC) and arterial blood gas parameters (e.g., PaO_2_, PaCO_2_, pH) were not included. Incorporating comprehensive cardiorespiratory measurements in future research would provide a more robust evaluation of physiological responses to the intervention.

## 5. Conclusions

The lung vibration resulted in higher sputum volume compared with conventional manual vibration, without evidence of immediate adverse physiological effects under short-term monitoring. These preliminary findings support its potential as an alternative in chest physiotherapy. Future studies should investigate long-term clinical benefits and explore its effectiveness in larger patient populations.

## Figures and Tables

**Table 1:** Baseline clinical characteristics of patients (*n* = 25).

Characteristic	Value
Age (years)	71.21 ± 6.24
Sex (*n*, %)	
Male	6 (24)
Female	19 (76)
BMI (kg/m^2^)	24.97 ± 4.28
Smoking status (*n*, %)	
- Current	0 (0)
- Ex-smoker	5 (20)
- Never smoker	20 (80)
Medications (*n*, %)	
- Antibiotic therapy	8 (32)
- Bronchodilator therapy	19 (76)
- Corticosteroid therapy	1 (4)
Using supplementary oxygen (*n*, %)	10 (40)
Length of hospital admission (days)	8.76 ± 1.98
Medical diagnosis	
- Bronchiectasis (*n*, %)	11 (44)
- Pneumonia (*n*, %)	8 (32)
- COPD with Bronchiectasis (*n*, %)	5 (20)
- COPD with Respiratory failure (*n*, %)	1 (4)

Data are presented as mean ± standard deviation (SD), frequencies, percentages. BMI, body mass index. COPD, chronic obstructive pulmonary disease.

**Table 2:** Sputum quantity and quality, dyspnea score, and peripheral oxygen saturation level of patients in the conventional and experimental interventions at pre- and post-intervention (*n* = 25).

Parameter	Conventional Intervention			Experimental Intervention			*p*-Value (Post vs. Post) with 95% CI
	Pre	Post	*p*-Value	Pre	Post	*p*-Value	
Sputum							
Volume (mL)	-	3.31 ± 1.39	-	-	4.69 ± 2.51 **	-	0.005 (0.41, 2.34)
Type	-	2.17 ± 0.76	-	-	1.86 ± 0.74	-	0.107 (−0.07, 0.69)
Pourability score	-	2.44 ± 0.56	-	-	2.64 ± 0.61	-	0.061 (−0.87, 1.05)
RPD score	9.76 ± 1.30	10.31 ± 1.04	0.241	9.96 ± 1.59	10.44 ± 1.72	0.173	0.357 (−1.63, 0.30)
SpO_2_ (%)	96.50 ± 0.54	99.50 ± 0.71	0.374	95.93 ± 0.51	99.12 ± 0.91 *	<0.001	0.701 (−0.55, 0.82)

Data are presented as mean ± standard deviation. CI, confidence interval; RPD, rating of perceived dyspnea; SpO_2_, peripheral oxygen saturation. * *p* < 0.05 vs. Pre intervention; ** *p* < 0.05 vs. Conventional intervention.

**Table 3:** Period-specific sputum quantity and quality, dyspnea score, and peripheral oxygen saturation level of patients in the conventional and experimental interventions at post-intervention.

Parameter	Period 1		Period 2		*p*-Value (Period Effect)	*p*-Value (Sequence Effect)
	Conventional (*n* = 13)	Experimental (*n* = 12)	Conventional (*n* = 12)	Experimental (*n* = 13)		
Volume (mL)	3.07 ± 1.28	4.86 ± 2.54	3.57 ± 1.50	4.53 ± 2.56	0.854	0.450
Type	2.33 ± 0.72	1.64 ± 0.63	2.00 ± 0.78	2.07 ± 0.80	0.805	0.058
Pourability score	2.52 ± 0.47	2.61 ± 0.59	2.38 ± 0.51	2.68 ± 0.54	0.721	0.339
RPD score	11.33 ± 1.18	10.07 ± 1.82	11.29 ± 0.91	10.85 ± 1.57	0.316	0.257
SpO_2_ (%)	99.71 ± 0.63	99.08 ± 0.82	99.39 ± 0.78	99.23 ± 0.96	0.510	0.851

Data are presented as mean ± standard deviation. RPD, rating of perceived dyspnea; SpO_2_, peripheral oxygen saturation.

**Table 4:** Cardiovascular dynamics, respiratory rate, and body temperature level of patients in the conventional and experimental interventions at pre- and post-intervention (*n* = 25).

Parameter	Conventional Intervention			Experimental Intervention			*p*-Value (Post vs. Post) with 95% CI
	Pre	Post	*p*-Value	Pre	Post	*p*-Value	
HR (bpm)	95.10 ± 19.41	101.93 ± 18.52 *	<0.001	91.81 ± 14.19	98.96 ± 13.99 *	<0.001	0.673 (−3.14, 2.03)
SBP (mmHg)	130.59 ± 12.28	132.00 ± 11.35	0.197	126.18 ± 11.87	132.37 ± 9.67 *	0.003	0.608 (−0.14, 6.25)
DBP (mmHg)	76.83 ± 9.35	77.38 ± 8.22	0.581	75.36 ± 10.89	76.22 ± 9.22	0.719	0.582 (−2.30, 1.90)
PP (mmHg)	53.76 ± 16.72	54.62 ± 14.33	0.487	50.82 ± 11.13	56.15 ± 12.39 *	0.007	0.511 (−0.18, 7.19)
MAP (mmHg)	94.75 ± 6.82	95.59 ± 6.51	0.324	92.30 ± 9.93	94.94 ± 7.33	0.050	0.427 (−1.16, 2.73)
RPP × 10^−2^ (mmHg·bpm)	123.68 ± 25.25	134.19 ± 24.91 *	<0.001	116.37 ± 24.47	131.46 ± 23.36 *	<0.001	0.334 (−2.62, 7.72)
RR (/min)	20.83 ± 2.17	21.14 ± 2.10	0.071	21.00 ± 2.09	20.78 ± 2.42	0.641	0.304 (−1.48, 0.46)
BT (°C)	36.99 ± 0.64	37.11 ± 0.59	0.932	36.76 ± 0.45	37.06 ± 0.48	0.626	0.312 (−0.00, 0.01)

Data are presented as mean ± standard deviation. BT, body temperature; CI, confidence interval; DBP, diastolic blood pressure; HR, heart rate; MAP, mean arterial pressure; PP, pulse pressure; RPP, rate–pressure product; RR, respiratory rate; SBP, systolic blood pressure. * *p* < 0.05 vs. Pre intervention.

**Table 5:** Period-specific cardiovascular dynamics, respiratory rate, and body temperature level of patients in the conventional and experimental interventions at post-intervention.

Parameter	Period 1		Period 2		*p*-Value (Period Effect)	*p*-Value (Sequence Effect)
	Conventional (*n* = 13)	Experimental (*n* = 12)	Conventional (*n* = 12)	Experimental (*n* = 13)		
HR (bpm)	103.00 ± 19.07	106.14 ± 14.20	100.79 ± 18.56	91.23 ± 8.97	0.016	0.193
SBP (mmHg)	130.07 ± 10.46	131.57 ± 8.41	134.07 ± 12.29	133.23 ± 11.16	0.244	0.700
DBP (mmHg)	79.00 ± 6.62	77.36 ± 9.15	75.64 ± 9.60	75.00 ± 9.50	0.118	0.868
PP (mmHg)	51.07 ± 13.50	54.21 ± 11.19	58.43 ± 14.70	58.23 ± 13.71	0.037	0.691
MAP (mmHg)	96.02 ± 5.02	95.43 ± 7.18	95.12 ± 7.98	94.41 ± 7.74	0.540	0.965
RPP × 10^−2^ (mmHg·bpm)	133.53 ± 24.27	140.55 ± 25.76	134.89 ± 26.47	121.66 ± 16.22	0.086	0.179
RR (/min)	20.67 ± 2.09	21.43 ± 1.83	21.64 ± 2.06	20.08 ± 2.84	0.743	0.052
BT (°C)	36.89 ± 0.68	36.71 ± 0.38	37.10 ± 0.60	36.81 ± 0.51	0.274	0.692

Data are presented as mean ± standard deviation. BT, body temperature; DBP, diastolic blood pressure; HR, heart rate; MAP, mean arterial pressure; PP, pulse pressure; RPP, rate–pressure product; RR, respiratory rate; SBP, systolic blood pressure.

**Table 6:** Potential adverse effects in response to conventional and experimental interventions (*n* = 25).

Adverse Effect	Post-Conventional Intervention	Post-Experimental Intervention
Breathlessness symptom (*n*, %)	0 (0)	0 (0)
Chest pain symptom (*n*, %)	0 (0)	0 (0)

Data are presented as frequencies, percentages.

## Data Availability

The data are available upon request from the corresponding author. Restrictions apply to the availability of these data due to privacy and ethical considerations.

## Abbreviations

The following abbreviations are used in this manuscript:

ANCOVA	Analysis of covariance
BMI	Body mass index
BT	Body temperature
CI	Confidence interval
COPD	Chronic obstructive pulmonary disease
COVID-19	Coronavirus disease 2019
DBP	Diastolic blood pressure
FEV	Forced expired volume in 1 s
FEV_1_/FVC	Ratio of forced expiratory volume in 1 s/forced vital capacity
FVC	Forced vital capacity
HR	Heart rate
ICU	Intensive care unit
MAP	Mean arterial pressure
PaCO_2_	Arterial partial pressure of carbon dioxide
PaO_2_	Arterial partial pressure of oxygen
PP	Pulse pressure
RPD	Rating of perceived dyspnea
RPP	Rate–pressure product
RR	Respiratory rate
SBP	Systolic blood pressure
SD	Standard deviation
SpO_2_	Oxygen saturation
